# Effects of number of training generations on genomic prediction for various traits in a layer chicken population

**DOI:** 10.1186/s12711-016-0198-9

**Published:** 2016-03-19

**Authors:** Ziqing Weng, Anna Wolc, Xia Shen, Rohan L. Fernando, Jack C. M. Dekkers, Jesus Arango, Petek Settar, Janet E. Fulton, Neil P. O’Sullivan, Dorian J. Garrick

**Affiliations:** Department of Animal Science, Iowa State University, Ames, IA 50010 USA; Hy-Line International, Dallas Center, IA 50063 USA; Department of Medical Epidemiology and Biostatistics, Karolinska Institute, Stockholm, Sweden; MRC Human Genetics Unit, MRC Institute of Genetics and Molecular Medicine, University of Edinburgh, Edinburgh, UK; Institute of Veterinary, Animal and Biomedical Sciences, Massey University, Palmerston North, New Zealand

## Abstract

**Background:**

Genomic estimated breeding values (GEBV) based on single nucleotide polymorphism (SNP) genotypes are widely used in animal improvement programs. It is typically assumed that the larger the number of animals is in the training set, the higher is the prediction accuracy of GEBV. The aim of this study was to quantify genomic prediction accuracy depending on the number of ancestral generations included in the training set, and to determine the optimal number of training generations for different traits in an elite layer breeding line.

**Methods:**

Phenotypic records for 16 traits on 17,793 birds were used. All parents and some selection candidates from nine non-overlapping generations were genotyped for 23,098 segregating SNPs. An animal model with pedigree relationships (PBLUP) and the BayesB genomic prediction model were applied to predict EBV or GEBV at each validation generation (progeny of the most recent training generation) based on varying numbers of immediately preceding ancestral generations. Prediction accuracy of EBV or GEBV was assessed as the correlation between EBV and phenotypes adjusted for fixed effects, divided by the square root of trait heritability. The optimal number of training generations that resulted in the greatest prediction accuracy of GEBV was determined for each trait. The relationship between optimal number of training generations and heritability was investigated.

**Results:**

On average, accuracies were higher with the BayesB model than with PBLUP. Prediction accuracies of GEBV increased as the number of closely-related ancestral generations included in the training set increased, but reached an asymptote or slightly decreased when distant ancestral generations were used in the training set. The optimal number of training generations was 4 or more for high heritability traits but less than that for low heritability traits. For less heritable traits, limiting the training datasets to individuals closely related to the validation population resulted in the best predictions.

**Conclusions:**

The effect of adding distant ancestral generations in the training set on prediction accuracy differed between traits and the optimal number of necessary training generations is associated with the heritability of traits.

**Electronic supplementary material:**

The online version of this article (doi:10.1186/s12711-016-0198-9) contains supplementary material, which is available to authorized users.

## Background

Genomic prediction in domestic animals is rapidly becoming the preferred method to evaluate individual genetic merit with advances in technology for massively parallel genotyping of SNPs (single nucleotide polymorphisms). Genomic selection is considered a promising approach, since it can yield higher rates of genetic gain and lower rates of inbreeding per generation than pedigree-based best linear unbiased prediction (PBLUP) [1, 2], which is the traditional approach for calculating estimated breeding values (EBV) based on phenotype and pedigree information [3]. Simulated and real data analyses have shown that accuracies of both genomic prediction and PBLUP can be influenced by the heritability of the trait, the nature of the fixed effects, and the extent of additive genetic relationships between phenotyped individuals and selection candidates [4]. Genomic prediction accuracies are affected by marker density [5], number of animals in the training population [6, 7], size and number of quantitative trait loci (QTL) [8, 9], and amount of linkage disequilibrium (LD) or linkage between markers and QTL [10]. Collectively, the latter two factors characterize the genomic architecture of the trait.

Based on simplistic theory, the larger is the number of animals used in training, the greater is the expected accuracy of genomic prediction [6, 7]. Inclusion of data on animals from past generations will increase the size of the training data set. As briefly described below, another reason for using data from all past generations is to avoid selection bias [11, 12]. Under random mating, the joint distribution between phenotypic and breeding values can be specified using the theory of covariance between relatives. This joint distribution is used to predict breeding values from phenotypes. In a population that is under selection, this joint distribution is altered in a way that depends on the type and intensity of selection and thus, prediction of breeding values becomes difficult. However, when inference is based on conditional distributions and conditioning is on data that includes all the information used for selection, it has been shown that the selection process can be ignored [12–14]. Pedigree-based additive genetic covariance between a candidate and its direct ancestor is halved by each additional generation. Thus, in PBLUP, under random mating, data from distant generations contribute little to the accuracy of prediction. In a simulated population under selection, it has been shown that using the data from the last two generations compared to that of the full pedigree resulted in the same response to selection [15]. This should be examined in a real population under selection. In contrast to PBLUP, in genomic BLUP (GBLUP), given the high LD between markers and QTL, even distant generations are expected to contribute to prediction accuracy [16]. Lourenco et al. [17] evaluated the benefit of past generations on the accuracy of GEBV using single-step GBLUP, where the genomic relationship matrix was blended with the pedigree-based relationship matrix. Using one set of individuals for validation, they found a small effect of pedigree depth on the accuracy of GEBV [17].

The objective of our study was to examine the effect of including successive generations in the training dataset on accuracy of genomic prediction across different validation sets and to assess the optimal number of training generations for routinely recorded traits. Using data from an elite line of layer chickens, genomic predictions were obtained by using the BayesB genomic prediction method [5] and PBLUP, and the resulting predictions were compared.

## Methods

### Phenotypes and genotypes

Data included phenotypic records for 17,793 birds from an experimental brown-egg laying population, representing 11 generations that hatched between 2002 and 2011. Among those, 5108 birds (including all parents used for breeding) from the most recent nine generations (from G3 to G11) were genotyped with a custom 40 K SNP panel (Illumina, San Diego, CA). Only genotyped females (~2260) with their own phenotypic records were used in the prediction analyses. A total of 23,098 segregating SNPs across 28 chromosomes remained after removing SNPs with a call rate lower than 0.95 (1121 SNPs), a minor allele frequency lower than 0.025 (10,770 SNPs), or a Mendelian inconsistency rate between parent-offspring higher than 0.05 (1467 SNPs). The following 16 traits were analyzed: early and late albumen height (eAH, lAH, mm), shell color of the first three eggs (eC3, index units), weight of the first three eggs (eE3, g), early and late egg color (eCO, lCO, index units), early and late average egg weight (eEW, lEW, g), early and late egg production rate (ePD, lPD), early and late shell puncture score (ePS, lPS, g/s), early and late yolk weight (eYW, lYW, g), body weight (lBW, kg) and age at sexual maturity (eSM, d). Measurements of early and late traits were taken at 26–28 and 42–46 weeks, respectively, except for eC3 and eE3, which were measured when hens reached sexual maturity. In total, there were 136,243 and 45,242 phenotypic records for early and late traits, respectively. The pedigree-based heritability (narrow-sense heritability *h*^*2*^) for each trait was estimated by using a single-trait animal model fitted in ASREML [18] for all phenotyped animals. In this selection program, genomic information was used since 2009 (G7, generation 7), after many generations of conventional multiple-trait selection based on an index of EBV [19]. Three hundred and sixty females and 120 males (out of ~2000 birds) were selected per generation during conventional selection, whereas when genomic selection started, 50 animals of each sex (out of ~600 birds) were selected from G7 to G11. The basic description of the collected phenotypic records is in Table 1.

**Table 1:** Summary statistics of the phenotypes available for 16 traits^a^ in each generation (G)

Gen		eCO	eEW	eC3	eE3	eSM	eAH	eYW	ePD	ePS	lCO	lEW	lBW	lAH	lYW	lPD	lPS
G1	N	436	436	410	410	440	436	0	440	436	414	414	440	414	0	414	411
	Mean	70.9	56.3	69.0	45.4	153.6	7.3	0	81.1	1604.9	59.4	62.1	2.1	5.6	0	67.5	1383.5
	SD	9.1	4.5	9.1	4.7	11.1	0.8	0	12.9	49.4	10.1	4.9	0.3	0.9	0	14.3	30.6
G2	N	1669	1669	1667	1667	1669	1667	1657	1669	1668	588	588	588	586	582	588	588
	Mean	69.6	55.2	70.6	44.3	151.9	7.1	14.9	81.6	1509.0	66.4	60.5	2.0	6.5	17.9	77.7	1601
	SD	8.5	4.8	8.6	4.7	8.5	0.9	1.7	12.4	93.2	8.5	4.7	0.3	0.9	1.5	14.5	59.2
G3	N	2738	2737	2729	2729	2738	2737	2728	2738	2738	649	649	647	649	646	635	649
	Mean	73.3	56.8	74.6	43.6	149.3	7.1	15.2	80.9	1425.0	72.4	61.5	2.0	6.6	17.8	77.3	1435.4
	SD	7.7	4.6	7.9	4.5	7.4	1.0	1.1	11.3	38.4	7.6	4.6	0.3	0.9	1.2	12.1	25.0
G4	N	2771	2772	2753	2752	2772	2771	2736	2772	2770	794	794	793	794	793	784	794
	Mean	71.4	57.5	74.4	46.7	156.3	7.5	15.1	82.4	1388.3	66.9	62.2	2.0	7.2	17.8	80.6	1399.8
	SD	8.2	4.8	7.7	5.1	9.9	1.0	1.1	11.3	39.9	9.3	4.5	0.2	0.9	1.3	12.1	40.6
G5	N	2964	2964	2952	2951	2964	2963	2958	2965	2964	782	782	781	782	781	778	782
	Mean	76.1	58.0	75.4	47.3	159.8	7.4	15.3	84.9	1494.9	72.9	63.5	2.0	7.2	18.1	82.4	1508.6
	SD	7.5	4.9	7.9	4.6	6.2	1.0	1.2	9.8	42.5	7.9	4.7	0.3	0.9	1.4	11	36.4
G6	N	2117	2117	2103	2103	2117	2116	2115	2117	2115	769	768	759	769	768	755	769
	Mean	77.2	57.2	78.1	45.2	147.6	7.4	15.1	83.3	1459.9	70.9	62.7	1.8	6.9	18.1	80.0	1496.1
	SD	7.7	4.9	7.9	4.7	7.8	1.0	1.2	10.3	42.8	8.6	4.8	0.3	0.9	1.4	11.0	36.6
G7	N	290	290	278	278	290	290	290	290	289	280	280	277	280	275	274	280
	Mean	78.1	59.2	80.2	45.0	148.9	7.7	15.4	83.1	1492.6	71.6	63.3	1.8	7.5	17.9	77.4	1487.8
	SD	7.3	4.8	7.6	4.5	7.8	1.1	1.1	9.2	41.7	8.6	4.9	0.3	0.9	1.4	11.7	35.0
G8	N	251	252	275	275	272	252	250	263	249	270	270	271	270	263	262	268
	Mean	80.5	56.8	79.8	44.2	142.0	7.9	15.2	80.8	1451.6	71.7	61.2	1.8	7.4	17.8	76.9	1423.9
	SD	7.5	4.9	7.5	4.6	5.6	1.1	1.35	8.0	39.8	8.1	4.9	0.3	1.1	1.5	11.0	35.9
G9	N	300	300	299	299	302	300	296	302	300	292	292	294	292	285	291	292
	Mean	79.2	59.1	82.5	44.1	141.9	8.4	15.9	83.2	1498.6	78.8	61.8	2.0	8.1	17.4	78.8	1519.2
	SD	7.7	4.5	7.7	4.7	7.6	1.0	1.18	10.1	37.6	8.3	5.1	0.2	1.1	1.5	10.1	39.1
G10	N	724	724	828	828	850	723	708	850	723	835	829	850	835	828	826	832
	Mean	83.3	58.6	79.3	46.2	146.5	8.0	14.9	87.9	1474.9	75.8	62.8	2.0	8	17.5	80.0	1471.6
	SD	8.0	4.4	7.8	5.0	7.9	1.1	1.19	9.4	45.9	7.8	4.5	0.2	1.1	1.3	11.7	49.4
G11	N	899	899	891	891	899	899	898	899	896	856	856	867	856	850	899	855
	Mean	83.3	56.4	82.7	44.7	139.2	8.6	14.3	81.7	1514.1	77.8	61.0	2.0	7.7	17.3	77.6	1403.2
	SD	8.2	4.5	7.2	4.7	7.8	0.9	1.1	10.0	19.2	8.9	4.5	0.2	0.9	1.3	9.5	27.3

^a^Early (e) and late (l) CO (egg color, index units), EW (average weight of 3 to 5 eggs, g), C3 (color of first 3 eggs, index units), E3 (weight of first 3 eggs, g), AH (albumen height, mm), PD (egg production rate), PS (puncture score, g/s), and YW (yolk weight, g); eSM (age at sexual maturity, d); lBW (body weight, kg)

### Statistical models

The following two single-trait models were used to predict EBV or GEBV:

1. PBLUP: a single-trait animal model using pedigree relationships and all available phenotype records was fitted using ASREML3.0 [18]. The model equation was:
  $$ {\mathbf{y}} = {\mathbf{X{\varvec\upbeta} }} + {\mathbf{Za}} + {\mathbf{e}}, $$

where **y** is the vector of phenotypes for each trait in the training set, ${\varvec{\upbeta}}$ represents the vector of fixed class effects (hatch within generation), ${\mathbf{a}}\varvec{ }$ is the vector of animal breeding values with $Var\left( {\mathbf{a}} \right) = {\mathbf{A}}\sigma_{a}^{2} ,$ where ${\mathbf{A}}$ is the pedigree relationship matrix and $\sigma_{a}^{2}$ is the additive genetic variance estimated using ASREML, ${\mathbf{X}}$ and ${\mathbf{Z}}$ are design matrices, and ${\mathbf{e}}$ is the vector of residual effects with $Var\left( {\mathbf{e}} \right) = {\mathbf{I}}\sigma_{e}^{2}$, where $\sigma_{e}^{2}$ is the residual variance estimated using ASREML. In the pedigree-based analyses, the relationship matrix was calculated from either the full pedigree including all animals from 11 generations, or from truncated pedigrees that only included ancestors that were born within two generations prior to the training set. By solving the following mixed model equation [11], the EBV of individuals in the validation population, whose phenotypes were masked, were obtained:

$$ \left[ {\begin{array}{*{20}c} {{\mathbf{X}}^{{\prime }} {\mathbf{X}}} &\quad {{\mathbf{X}}^{{\prime }} {\mathbf{Z}}} \\ {{\mathbf{Z}}^{{\prime }} {\mathbf{X}}} &\quad {{\mathbf{Z}}^{{\prime }} {\mathbf{Z}} + {\mathbf{A}}^{ - 1} \lambda } \\ \end{array} } \right]\left[ {\begin{array}{*{20}c} {{\hat{\varvec{\upbeta }}}} \\ {{\hat{\mathbf{a}}}} \\ \end{array} } \right] = \left[ {\begin{array}{*{20}c} {{\mathbf{X}}^{{\prime }} {\mathbf{y}}} \\ {{\mathbf{Z}}^{{\prime }} {\mathbf{y}}} \\ \end{array} } \right], $$

where $\lambda = \sigma_{e}^{2} /\sigma_{a}^{2}$, ${\hat{\varvec{\upbeta }}}$ is the vector of estimates of fixed class effects, and ${\hat{\mathbf{a}}}$ is the vector of EBV of animals included in the full or truncated pedigrees.

(2) Genomic prediction model BayesB [5, 20] was applied using only records on genotyped individuals that had their own phenotypic records (i.e. only females) and was performed using the GenSel4.4 software [20, 21]. Method BayesB assumes that a fraction $\pi$ of SNPs have zero effects and 1-$\pi$ SNP effects have a univariate-t distribution with a mean of 0, $v_{a}$ degrees of freedom, and a scale parameter $S_{a}^{2}$. This prior assumption of SNP effects is equivalent to assuming that each SNP effect has a univariate normal distribution with a mean of 0 and a SNP-specific variance [22]. Each SNP-specific variance has a scaled inverse Chi square prior distribution with $v_{j}$ = 4.2 degrees of freedom and a scale parameter $S_{j}^{2}$ derived from $\frac{{\tilde{\sigma }_{j}^{2} \left( {v_{j} - 2} \right)}}{{v_{j} }}$, where $\tilde{\sigma }_{j}^{2}$ is the variance of the additive effect for a randomly sampled SNP calculated as $\frac{{\tilde{\sigma }_{s}^{2} }}{{\left( {1 - \pi } \right)\mathop \sum \nolimits_{j = 1}^{k} 2p_{j} \left( {1 - p_{j} } \right)}}$, where $\tilde{\sigma }_{s}^{2}$ is the additive genetic variance explained by SNPs, and $p_{j}$ is the allele frequency of SNP $j$ [22]. The priors for the genetic and residual variances for each trait were obtained from the single-trait pedigree-based ASREML analyses. Markov chain Monte Carlo (MCMC) sampling with 55,000 iterations, of which the first 5000 were discarded as burn-in, was used to estimate the posterior means of SNP effects. The convergence of MCMC samples for genetic variance, residual variance, and marker heritability were assessed by using the Heidelberger and Welch test [23] in R/coda package [24]. The model equation used for BayesB is:

$$ y_{im} = \beta_{m} + \mathop \sum \limits_{j = 1}^{k} z_{ij} u_{j} + e_{i} , $$

where $y_{im}$ is the phenotype for genotyped individual *i* in the training set in hatch within generation class *m*, $\beta_{m}$ is the effect of hatch within generation class *m*, *k* is the number of SNPs, $z_{ij}$ is the allele at SNP *j* in genotyped individual *i* coded 0, 1 and 2, $u_{j}$ is the random effect of SNP *j* distributed as $u_{j} \;\sim\;N\left( {0,\sigma_{j}^{2} } \right)$ with probability $1 - \pi$, and 0 otherwise, where $\sigma_{j}^{2}$ is the variance of the additive effect for SNP *j*, and $e_{i}$ is the residual effect distributed as $e_{i} \;\sim\;N\left( {0,\sigma_{e}^{2} } \right)$, where $\sigma_{e}^{2}$ is the residual variance. The assumed value of $\pi$ was 0.95. The GEBV of individual *i* ($GEBV_{i}$) in the validation population was derived as:

$$ GEBV_{i} = \mathop \sum \limits_{j = 1}^{k} z_{ij} \hat{u}_{j} , $$

where $z_{ij}$ is the allele at SNP *j* of the genotyped individual *i*, and $\hat{u}_{j}$ is the posterior mean of the substitution effect of SNP *j* estimated over 50,000 post burn-in samples.

The effect of using different training generations, including animals with phenotypes and genotypes (~300 per generation), was assessed for generations G5–G11. The training sets consisted of animals from successive ancestral generations immediately prior to the validation generation. Additional file 1: Table S1 uses an example to illustrate the assignment of validation and training sets. Different validation sets (from G5 to G11) with different numbers of training generations were assessed. If only G11 was used for validation, spurious environmental effects, such as heat stress in a particular year, would be confounded with the distance between the training and validation generations, which could bias results. Thus, different validation generations were used to avoid this confounding. The maximum numbers of training generations for pedigree-based and marker-based analyses were 10 and 8, respectively. The numbers of phenotypic records within each generation are in Table 1. Additional file 1: Table S2 gives the average number of available genotyped individuals with early and late traits for each generation. Predictive performance of each model was evaluated by prediction accuracy, which was determined in the validation generation based on the correlation between EBV and phenotypes adjusted for fixed effects, standardized by dividing by the square root of trait heritability [25, 26].

In order to separate the impact of size of the training data set and number of training generations on prediction accuracy of GEBV, additional training scenarios were considered for one of the analyzed traits as an example (eEW) using the BayesB model (Table 2). In that analysis, G10 was used as the validation set and different numbers of genotyped animals (125 or 250) were randomly sampled from one to six training generations (G4–G9). The training scenarios differed in total number of animals and number of generations that contributed to the training set. Some scenarios had the same size of training set but differed in the number of generations that contributed to the training set. For example, scenarios 1 and 5 had 250 genotyped animals in the training set, but in scenario 1, all these 250 animals were from G9, whereas in scenario 5, 125 animals were from G8 and the remaining 125 animals were from G9. Each scenario was repeated five times in order to avoid sample bias.

**Table 2:** Mean accuracy (±SD) of genomic predictions over 5 replicates obtained with different training sets^a^ for eEW^b^

Scenario	Distribution of training animals across generations	Number of generations in training	Number of animals in training	Prediction accuracy (±SD)
1	G9 = 250	1	250	0.46 ± 0.089
2	G9 = G8 = 250	2	500	0.60 ± 0.019
3	G9 = G8 = G7 = 250	3	750	0.64 ± 0.017
4	G9 = 125	1	125	0.23 ± 0.021
5	G9 = G8 = 125	2	250	0.45 ± 0.088
6	G9 = G8 = G7 = 125	3	375	0.57 ± 0.038
7	G9 = G8 = G7 = G6 = 125	4	500	0.57 ± 0.021
8	G9 = G8 = G7 = G6 = G5 = 125	5	625	0.58 ± 0.010
9	G9 = G8 = G7 = G6 = G5 = G4 = 125	6	750	0.58 ± 0.013

^a^In this analysis, G10 was used as the validation generation and training individuals were randomly sampled from G4 to G9

^b^eEW, early average weight of 3–5 eggs

### Optimal number of training generations

The optimal number of training generations to maximize prediction accuracy was derived for each trait and method as the maximum from a second-order polynomial regression fitted to all the prediction accuracies that were obtained for that method for that trait, using the following model:

$$ y_{ik} = a_{i} k^{2} + b_{i} k + c_{i} + e_{ik} , $$

where $y_{ik}$ is the prediction accuracy of GEBV obtained using BayesB for trait $i$ with $k$ ancestral generations included in the training set, $a_{i}$ and $b_{i}$ are regression coefficients, $c_{i}$ is the intercept, and $e_{ik}$ is the residual. Significance of regression coefficients was tested for each trait. For all traits, except eCO, eC3, eSM, lAH, lYW, and lPS, the second-order polynomial regression coefficients were significantly (p < 0.01) different from zero. The optimal number of training generations was then derived as min $\left( { - \frac{{\hat{b}_{i} }}{{2\hat{a}_{i} }},8} \right)$ because the dataset included at most eight generations.

### Marker-based heritability

Marker-based heritability ($h_{q}^{2}$) was defined as the genetic variance explained by the markers divided by the total phenotypic variance. Genomic prediction method BayesC with π = 0 implemented in the GenSel4.4 software [20, 21] was used to estimate $h_{q}^{2}$, which assumes that all the SNPs have non-zero effects, and each SNP effect is drawn from a normal distribution with a common variance. This BayesC0 model is equivalent to GBLUP [27], except that genetic and residual variances are treated as unknown with given priors, instead of being fixed in GBLUP. The priors for the genetic and residual variance components were obtained from the single-trait pedigree-based ASREML analysis for each trait. MCMC sampling with 55,000 iterations (discarding the first 5000 as burn-in) was used to make inference on $h_{q}^{2}$.

## Results and discussion

### Prediction accuracy in progeny

#### Differences between prediction methods

**Fig. 1:**
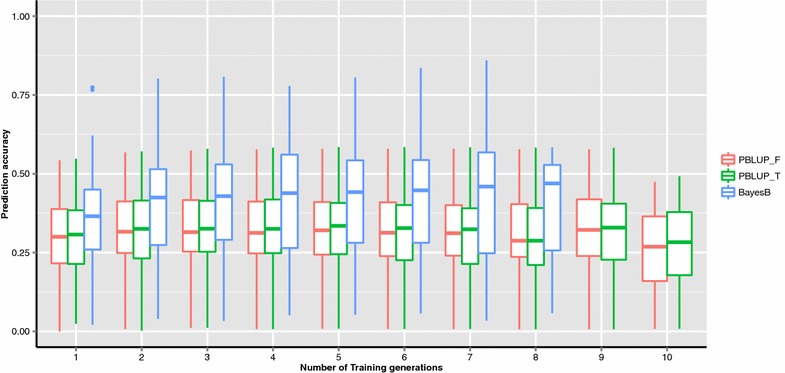
Prediction accuracies of EBV over different numbers of training generations across all traits and all validation sets using genomic prediction (BayesB) or pedigree-based BLUP with a truncated (PBLUP_T), or full pedigree (PBLUP_F). The full pedigree included all animals from 11 generations; the truncated pedigree included training and validation animals and their relatives traced two generations back. The *bar* *within each* *box* represents the median of prediction accuracies

Figure 1 shows boxplots of the prediction accuracies of PBLUP and BayesB for different training generations. Prediction accuracies of PBLUP quickly reached a plateau as the number of training generations increased. The slight fluctuations in prediction accuracies of PBLUP might be due to genetic drift. Prediction accuracies of PBLUP using a truncated pedigree (PBLUP_T, including animals in the training and validating sets, and their relatives that were traced two generations back) were very similar compared to the full pedigree (PBLUP_F, including all animals from 11 generations) across validation generations. These results indicate that using a truncated or full pedigree to construct the pedigree-based relationship matrix has no significant effect on the accuracy of PBLUP in terms of ranking the current cohort of candidates in this population, which was under selection. Mehrabani-Yeganeh et al. [15] reported that using only the last two generations compared to the full pedigree resulted in the same response to selection in a simulated closed nucleus broiler line. Lourenco et al. [17] also found that depth of pedigree had a very small impact on the accuracy of PBLUB evaluations in US dairy cattle and pig data. They observed the same result for accuracies of GEBV using single-step GBLUP.

In our data, the advantage of genomic evaluations using BayesB over the pedigree-based EBV was obvious (Fig. 1) and can explained by the fact that genomic prediction uses LD between markers and QTL, as well as pedigree relationships [16]. Prediction accuracies obtained from PBLUP reached a plateau much more quickly as the number of training generations increased than those obtained from BayesB, because pedigree-based relationships decay faster than genomic relationships [4, 16].

In this study, MCMC samples from BayesB for genetic variance, residual variance, and marker-based heritability had converged based on Heidelberger and Welch diagnostics. A fixed π (0.95) was used in the BayesB analyses for all traits. Although using π estimated with the Bayes Cπ method [22] may result in different prediction for some analyses, using a fixed π in the BayesB analysis is not expected to affect the comparison of results within a trait. The BayesB method used in this study uses only animals with known phenotypes and genotypes. In contrast, single-step GBLUP uses pedigree relationships to include phenotypes of non-genotyped individuals.

#### Differences between traits and training generations

**Fig. 2:**
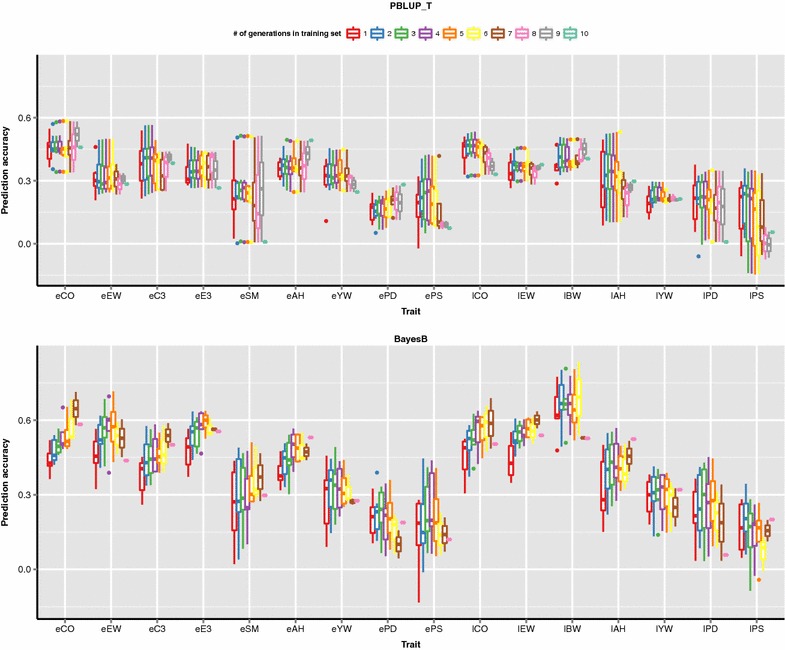
Prediction accuracies of EBV across different validation sets using pedigree BLUP with ancestors traced back two generations (PBLUP_T) and genomic prediction over different numbers of training generations for each trait. The *bar within each box* represents the median of prediction accuracies

In general, for the first few training generations, prediction accuracies of PBLUP and BayesB increased and then plateaued or dropped slightly when adding more distant ancestral generations (Fig. 2). The impact of adding ancestor generations in the training set on prediction accuracy of GEBV differed between traits. These differences might be caused by differences in heritabilities, genetic architecture, and the number of available genotypes or phenotypes. For some traits (e.g. eAH), prediction accuracy continued to increase as the number of training generations increased, while for other traits accuracies decreased slightly as the number of distant generations in training set increased (e.g. eEW).

In this population, data from distant generations (more than four training generations back) contributed little to prediction accuracy of PBLUP. For most traits, distant ancestral generations continued to contribute to the accuracy of genomic prediction but their contributions were smaller than those of generations that were close to the validation generation. For the same population, Wolc et al. [28] reported that decreasing the genomic relationships between pairs of individuals when the pedigree relationship was less than 0.45, effectively reduced the impact of distant relatives, and increased prediction accuracy for egg production in laying hens when using GBLUP.

To avoid confounding between environmental effects (e.g. heat stress) that can cause animals to re-rank and that might be specific to a particular generation, different validation sets were used in this study. We observed fluctuations in prediction accuracies over training generations, which could be due to variation in environmental effects, distinct population structures, different genomic relationships between training and validation sets, genetic drift, or interactions between genotype and environment. For example, in Additional file 2: Figure S1, the prediction accuracies of eEW ranged from 0.39 to 0.69 for different combinations of training and validation sets that were all characterized by having four generations included in the training set.

In this study, the size of the validation set, number of generations, and density of the SNP panel were limited by available data. Further analyses are needed to validate the effect on genomic prediction accuracy of adding distant ancestral generations in the training set. A larger population could allow the impact of these factors to be characterized and to better identify the contribution of each ancestral generation.

#### Size and composition of training set

Table 2 presents the prediction accuracies for eEW for eight scenarios that differed in the total number of training animals and the number of generations that contributed to the training set. As expected, for the same number of training generations, prediction accuracies increased with the size of the training set [6, 7]. For example, when the number of training animals from the same generation increased from 125 (scenario 4) to 250 (scenario 1), prediction accuracy of GEBV for the validation animals (G10) increased from 0.23 to 0.46.

Although the numbers of animals in the training set were the same between scenarios 2 and 7, prediction accuracy was greater in scenario 2 than in scenario 7 (Table 2). This difference was more obvious when the size of the training set became larger (comparing scenarios 3 and 9). In scenario 3, all 750 training animals were from the three preceding generations, whereas in scenario 9, 50 % of the animals were from more distant generations. Individuals from closely-related generations can better predict GEBV of validation animals compared to animals from more distant generations [16, 28]. Similar phenomena were observed for the 15 other traits (See Additional file 1: Table S3), except for ePD and lYW, for which prediction accuracy actually decreased as more animals from ancestral generations were added in the training set.

The number of genotyped animals per generation is limited in livestock species. Although increasing the number of training generations is not equivalent to increasing the size of the training set, including data from successive ancestral generations is an alternative approach to enlarge the size of the training population. However, the impact of including such ancestral generations in the training set on genomic prediction accuracies can differ between traits.

### Relationship between optimal number of training generations and heritability

Table 3 presents estimates of pedigree-based heritability and marker-based heritability for each trait. Marker-based heritabilities were smaller than pedigree-based heritabilities because markers did not capture all genetic variation.

**Table 3:** Estimates of pedigree-based and marker-based heritabilities (±SE) for the 16 traits^a^ from univariate animal models

Early traits	eCO	eEW	eC3	eE3	eSM	eAH	eYW	ePD	ePS
Pedigree-*h* ^b^	0.71 ± 0.017	0.69 ± 0.017	0.65 ± 0.018	0.61 ± 0.018	0.54 ± 0.018	0.51 ± 0.018	0.46 ± 0.019	0.34 ± 0.019	0.21 ± 0.015
Marker-*h* ^b^	0.55 ± 0.013	0.53 ± 0.013	0.47 ± 0.015	0.44 ± 0.014	0.31 ± 0.015	0.36 ± 0.015	0.30 ± 0.017	0.16 ± 0.017	0.15 ± 0.018

Late traits	lCO	lEW	lBW	lAH	lYW	lPD	lPS		
Pedigree-*h* ^b^	0.68 ± 0.025	0.61 ± 0.026	0.56 ± 0.026	0.48 ± 0.027	0.46 ± 0.028	0.25 ± 0.025	0.20 ± 0.028		
Marker-*h* ^b^	0.58 ± 0.019	0.50 ± 0.018	0.48 ± 0.020	0.34 ± 0.021	0.37 ± 0.020	0.19 ± 0.021	0.10 ± 0.024		

^a^Early (e) and late (l) CO (egg color, index units), EW (average weight of 3–5 eggs, g), C3 (color of first 3 eggs, index units), E3 (weight of first 3 eggs, g), AH (albumen height, mm), PD (egg production rate), PS (puncture score, g/s), and YW (yolk weight, g); eSM (age at sexual maturity, d); lBW (body weight, kg)

^b^ *SE* standard error

**Fig. 3:**
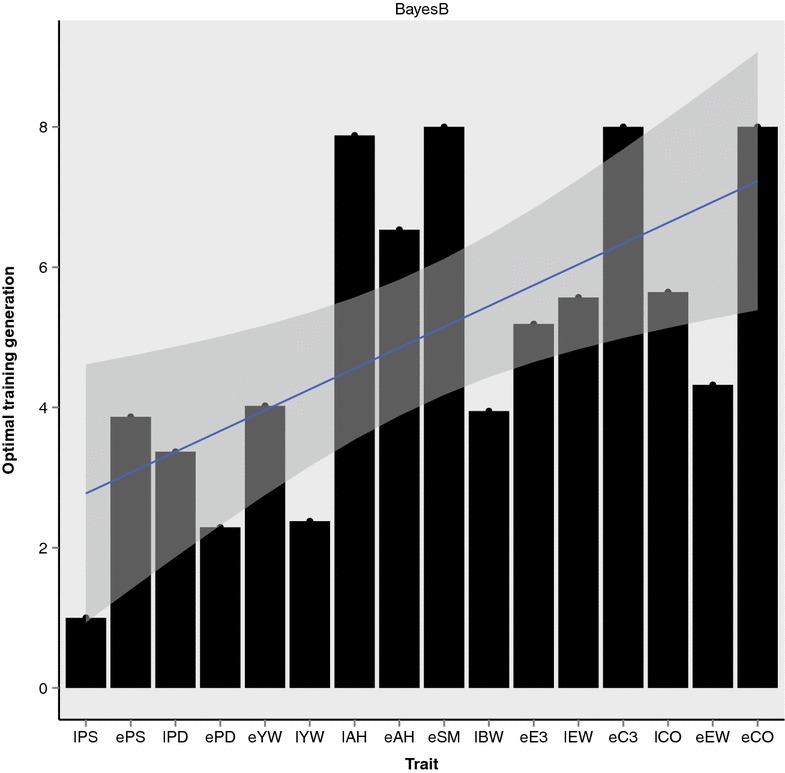
Optimal number of training generations for genomic prediction for each trait. Traits were sorted by pedigree-based heritability estimates. The *blue line* is the regression of the optimal number of training generations on heritability

Figure 3 shows the number of training generations that generated the highest accuracy of GEBV for each trait using BayesB. Traits were sorted by pedigree-based heritability estimates, from low (lPS) to high (eCO). The blue line in Fig. 3 shows the linear relationship between optimal training generation and pedigree-based heritability. The correlation between optimal number of training generations and pedigree-based heritability was equal to 0.65, whereas the correlation between optimal number of training generations and marker-based heritability was equal to 0.55. Additional file 2: Figure S1 shows in detail the regression of prediction accuracy on the number of training generations for each trait. In general, and somewhat surprisingly, the highly heritable traits had a larger optimal number of training generations than the lowly heritable traits.

Estimates of optimal number of training generations may vary according to assumptions of the statistical model and/or the density and location of SNPs. For some traits, if assumptions of the statistical model are not valid, the model may not capture the effects of QTL, even if the size of the training population increases. In a simulation study, Sun [29] showed that modeling co-segregation can improve prediction accuracy when the LD between SNPs and QTL is low in a training population that consisted of multiple families and generations. In the case where a causal variant or QTL is included in the SNP panel, adding data from more distant generations in the training set is expected to increase the accuracy of genomic prediction until the prediction accuracy reaches a plateau. When QTL mutations are not on the SNP panel, a high-density panel is likely to achieve higher LD since some SNPs will be closer to the QTL than would be the case with a low-density panel. Thus, when the dataset is sufficiently large and genotyped with high-density panels, the accuracy of genomic prediction is not expected to decrease when distant generations are used for the training set.

Based on this study, for highly heritable traits, prediction accuracy of GEBV was highest when the number of generations in the training set was larger than 4. In contrast, for lowly heritable traits, it was better to include in the training dataset only the individuals that were the most closely-related to the validation individuals. We suggest two strategies that may be useful for populations with multi-trait selection programs: (1) changing the number of training generations for each trait analyzed; or (2) obtaining a weighted optimal number of training generations based on results for all traits in the breeding objective. The weight for each trait could be determined by its relative economic importance in the breeding program.

## Conclusions

The effect of increasing the number of training generations on accuracy of genomic prediction differs between traits. The optimal number of training generations in genomic prediction is influenced by the heritability of a trait. For the data used in this study, traits with a lower heritability had a smaller optimal number of training generations than traits with a higher heritability. In practice, the optimal number of training generations to be used in a multi-trait selection population could be based on the importance of the traits in the breeding program.

## Additional files

10.1186/s12711-016-0198-9 Examples of experimental design for training (T) and validation (V) sets. **Table S2.** Description of the average number of individuals with own phenotypes and genotypes in each generation for early and late traits. **Table S3.** Mean accuracies (± SD) of genomic predictions over 5 replicates for different training sets and for the studied traits. In this analysis, G10 was used as the validation generation and training individuals were randomly sampled from G4 to G9. (1) eCO (early egg color); (2) eC3 (early color of first 3 eggs; (3) eE3 (early weight of first 3 eggs); (4) eSM (early age at sexual maturity); (5) eAH (early albumen height); (6) eYW (early yolk weight); (7) ePD (early egg production rate); (8) ePS (early egg puncture score); (9) lCO (late egge color); (10) lEW (late average weight of 3-5 eggs); (11) lBW (late body weight); (12) lAH (late albumen height); (13) lYW (late yolk weight); (14) lPD (late egg production rate); (15) lPS (late egg puncture score).
10.1186/s12711-016-0198-9 Scatter plot of accuracies of genomic predictions across different validation sets over training generations for each trait. The blue line is the regression of the accuracy on the number of training generations. The red line indicates the optimal number of training generations. The R-squared of regression line is presented as r2.
