# Supplementary material for: Effects of number of training generations on genomic prediction for various traits in a layer chicken population
Source: Genet Sel Evol. 2016 Mar 19;48:22. doi: 10.1186/s12711-016-0198-9 (PMC4799631; doi:10.1186/s12711-016-0198-9)
Supplement: Supplementary file 1 — 10.1186/s12711-016-0198-9 Examples of experimental design for training (T) and validation (V) sets. Table S2. Description of the average number of individuals with own phenotypes and genotypes in each generation for early and late traits. Table S3. Mean accuracies (± SD) of genomic predictions over 5 replicates for different training sets and for the studied traits. In this analysis, G10 was used as the validation generation and training individuals were randomly sampled from G4 to G9. (1) eCO (early egg color); (2) eC3 (early color of first 3 eggs; (3) eE3 (early weight of first 3 eggs); (4) eSM (early age at sexual maturity); (5) eAH (early albumen height); (6) eYW (early yolk weight); (7) ePD (early egg production rate); (8) ePS (early egg puncture score); (9) lCO (late egge color); (10) lEW (late average weight of 3-5 eggs); (11) lBW (late body weight); (12) lAH (late albumen height); (13) lYW (late yolk weight); (14) lPD (late egg production rate); (15) lPS (late egg puncture score). [file 12711_2016_198_MOESM1_ESM.docx]

**Table S1.** Examples of experimental design for training (T) and validation (V) sets.

| Generations | | | | | | | | | Generation used for validation | | Number of training generations  (from 1 to 8/10) | |  |
| --- | --- | --- | --- | --- | --- | --- | --- | --- | --- | --- | --- | --- | --- |
| … | G4 | G5 | G6 | G7 | G8 | G9 | G10 | G11 | |  | |  | |
| … |  |  |  |  |  |  | T | V | | G11 | | 1 | |
| … |  |  |  |  |  | T | V |  | | G10 | | 1 | |
| … |  |  |  |  | T | V |  |  | | G9 | | 1 | |
| … |  |  |  | T | V |  |  |  | | G8 | | 1 | |
| … |  |  |  |  |  | T | T | V | | G11 | | 2 | |
| … |  |  |  |  | T | T | V |  | | G10 | | 2 | |
| … |  |  |  | T | T | V |  |  | | G9 | | 2 | |
| … |  |  | T | T | V |  |  |  | | G8 | | 2 | |
| … |  |  |  |  | T | T | T | V | | G11 | | 3 | |
| … |  |  |  | T | T | T | V |  | | G10 | | 3 | |
| … |  |  | T | T | T | V |  |  | | G9 | | 3 | |
| … |  | T | T | T | V |  |  |  | | G8 | | 3 | |
| … |  |  |  | T | T | T | T | V | | G11 | | 4 | |
| … |  |  | T | T | T | T | V |  | | G10 | | 4 | |
| … |  | T | T | T | T | V |  |  | | G9 | | 4 | |
| … | T | T | T | T | V |  |  |  | | G8 | | 4 | |
| … | … | … | … | … | … | … | … | … | | … | | … | |

**Table S2.** Description of the average number of individuals with own phenotypes and genotypes in each generation for early and late traits.

| Generation | Number of genotyped with own records Early/Late |
| --- | --- |
| G1 | 0/0 |
| G2 | 0/0 |
| G3 | 295/295 |
| G4 | 322/323 |
| G5 | 295/295 |
| G6 | 360/357 |
| G7 | 287/278 |
| G8 | 260/268 |
| G9 | 300/291 |
| G10 | 240/277 |
| G11 | 300/290 |

**Table S3.** Mean accuracies (±SD) of genomic predictions over 5 replicates for different training sets for the studied traits. In this analysis, G10 was used as the validation generation and training individuals were randomly sampled from G4 to G9. (1) eCO (early egg color); (2) eC3 (early color of first 3 eggs; (3) eE3 (early weight of first 3 eggs); (4) eSM (early age at sexual maturity); (5) eAH (early albumen height); (6) eYW (early yolk weight); (7) ePD (early egg production rate); (8) ePS (early egg puncture score); (9) lCO (late egge color); (10) lEW (late average weight of 3-5 eggs); (11) lBW (late body weight); (12) lAH (late albumen height); (13) lYW (late yolk weight); (14) lPD (late egg production rate); (15) lPS (late egg puncture score).

1. eCO

| Scenario | Distribution of training animals across generations | Number of generations in training | Number of animals in training | Prediction accuracy (±SD) |
| --- | --- | --- | --- | --- |
| 1 | G9=250 | 1 | 250 | 0.42±0.076 |
| 2 | G9=G8=250 | 2 | 500 | 0.47±0.050 |
| 3 | G9=G8=G7=250 | 3 | 750 | 0.50±0.056 |
| 4 | G9=125 | 1 | 125 | 0.38±0.080 |
| 5 | G9=G8=125 | 2 | 250 | 0.40±0.046 |
| 6 | G9=G8=G7=125 | 3 | 375 | 0.41±0.029 |
| 7 | G9=G8=G7=G6=125 | 4 | 500 | 0.46±0.015 |
| 8 | G9=G8=G7=G6=G5=125 | 5 | 625 | 0.49±0.020 |
| 9 | G9=G8=G7=G6=G5=G4=125 | 6 | 750 | 0.50±0.027 |

1. eC3

| Scenario | Distribution of training animals across generations | Number of generations in training | Number of animals in training | Prediction accuracy (±SD) |
| --- | --- | --- | --- | --- |
| 1 | G9=250 | 1 | 250 | 0.28±0.038 |
| 2 | G9=G8=250 | 2 | 500 | 0.40±0.026 |
| 3 | G9=G8=G7=250 | 3 | 750 | 0.42±0.020 |
| 4 | G9=125 | 1 | 125 | 0.25±0.055 |
| 5 | G9=G8=125 | 2 | 250 | 0.26±0.050 |
| 6 | G9=G8=G7=125 | 3 | 375 | 0.38±0.018 |
| 7 | G9=G8=G7=G6=125 | 4 | 500 | 0.42±0.038 |
| 8 | G9=G8=G7=G6=G5=125 | 5 | 625 | 0.46±0.046 |
| 9 | G9=G8=G7=G6=G5=G4=125 | 6 | 750 | 0.46±0.051 |

1. eE3

| Scenario | Distribution of training animals across generations | Number of generations in training | Number of animals in training | Prediction accuracy (±SD) |
| --- | --- | --- | --- | --- |
| 1 | G9=250 | 1 | 250 | 0.35±0.021 |
| 2 | G9=G8=250 | 2 | 500 | 0.55±0.017 |
| 3 | G9=G8=G7=250 | 3 | 750 | 0.58±0.010 |
| 4 | G9=125 | 1 | 125 | 0.30±0.066 |
| 5 | G9=G8=125 | 2 | 250 | 0.42±0.10 |
| 6 | G9=G8=G7=125 | 3 | 375 | 0.51±0.062 |
| 7 | G9=G8=G7=G6=125 | 4 | 500 | 0.54±0.041 |
| 8 | G9=G8=G7=G6=G5=125 | 5 | 625 | 0.54±0.040 |
| 9 | G9=G8=G7=G6=G5=G4=125 | 6 | 750 | 0.52±0.040 |

1. eSM

| Scenario | Distribution of training animals across generations | Number of generations in training | Number of animals in training | Prediction accuracy (±SD) |
| --- | --- | --- | --- | --- |
| 1 | G9=250 | 1 | 250 | 0.39±0.016 |
| 2 | G9=G8=250 | 2 | 500 | 0.39±0.0093 |
| 3 | G9=G8=G7=250 | 3 | 750 | 0.39±0.0041 |
| 4 | G9=125 | 1 | 125 | 0.30±0.16 |
| 5 | G9=G8=125 | 2 | 250 | 0.33±0.099 |
| 6 | G9=G8=G7=125 | 3 | 375 | 0.31±0.11 |
| 7 | G9=G8=G7=G6=125 | 4 | 500 | 0.37±0.11 |
| 8 | G9=G8=G7=G6=G5=125 | 5 | 625 | 0.39±0.10 |
| 9 | G9=G8=G7=G6=G5=G4=125 | 6 | 750 | 0.38±0.11 |

1. eAH

| Scenario | Distribution of training animals across generations | Number of generations in training | Number of animals in training | Prediction accuracy (±SD) |
| --- | --- | --- | --- | --- |
| 1 | G9=250 | 1 | 250 | 0.28±0.060 |
| 2 | G9=G8=250 | 2 | 500 | 0.31±0.038 |
| 3 | G9=G8=G7=250 | 3 | 750 | 0.30±0.026 |
| 4 | G9=125 | 1 | 125 | 0.16±0.094 |
| 5 | G9=G8=125 | 2 | 250 | 0.24±0.11 |
| 6 | G9=G8=G7=125 | 3 | 375 | 0.20±0.11 |
| 7 | G9=G8=G7=G6=125 | 4 | 500 | 0.25±0.11 |
| 8 | G9=G8=G7=G6=G5=125 | 5 | 625 | 0.29±0.093 |
| 9 | G9=G8=G7=G6=G5=G4=125 | 6 | 750 | 0.31±0.083 |

1. eYW

| Scenario | Distribution of training animals across generations | Number of generations in training | Number of animals in training | Prediction accuracy (±SD) |
| --- | --- | --- | --- | --- |
| 1 | G9=250 | 1 | 250 | 0.15 ±0.037 |
| 2 | G9=G8=250 | 2 | 500 | 0.19 ±0.023 |
| 3 | G9=G8=G7=250 | 3 | 750 | 0.22 ±0.020 |
| 4 | G9=125 | 1 | 125 | 0.055 ±0.13 |
| 5 | G9=G8=125 | 2 | 250 | 0.15 ±0.11 |
| 6 | G9=G8=G7=125 | 3 | 375 | 0.18 ±0.086 |
| 7 | G9=G8=G7=G6=125 | 4 | 500 | 0.21±0.077 |
| 8 | G9=G8=G7=G6=G5=125 | 5 | 625 | 0.21 ±0.093 |
| 9 | G9=G8=G7=G6=G5=G4=125 | 6 | 750 | 0.22 ±0.11 |

1. ePD

| Scenario | Distribution of training animals across generations | Number of generations in training | Number of animals in training | Prediction accuracy (±SD) |
| --- | --- | --- | --- | --- |
| 1 | G9=250 | 1 | 250 | 0.067±0.049 |
| 2 | G9=G8=250 | 2 | 500 | 0.079±0.084 |
| 3 | G9=G8=G7=250 | 3 | 750 | 0.057±0.11 |
| 4 | G9=125 | 1 | 125 | 0.10±0.049 |
| 5 | G9=G8=125 | 2 | 250 | 0.11±0.084 |
| 6 | G9=G8=G7=125 | 3 | 375 | 0.10±0.11 |
| 7 | G9=G8=G7=G6=125 | 4 | 500 | 0.074±0.099 |
| 8 | G9=G8=G7=G6=G5=125 | 5 | 625 | 0.065±0.092 |
| 9 | G9=G8=G7=G6=G5=G4=125 | 6 | 750 | 0.064±0.094 |

1. ePS

| Scenario | Distribution of training animals across generations | Number of generations in training | Number of animals in training | Prediction accuracy (±SD) |
| --- | --- | --- | --- | --- |
| 1 | G9=250 | 1 | 250 | 0.13±0.018 |
| 2 | G9=G8=250 | 2 | 500 | 0.15±0.032 |
| 3 | G9=G8=G7=250 | 3 | 750 | 0.21±0.027 |
| 4 | G9=125 | 1 | 125 | 0.070±0.21 |
| 5 | G9=G8=125 | 2 | 250 | 0.10±0.20 |
| 6 | G9=G8=G7=125 | 3 | 375 | 0.12±0.24 |
| 7 | G9=G8=G7=G6=125 | 4 | 500 | 0.15±0.23 |
| 8 | G9=G8=G7=G6=G5=125 | 5 | 625 | 0.16±0.22 |
| 9 | G9=G8=G7=G6=G5=G4=125 | 6 | 750 | 0.15±0.23 |

1. lCO

| Scenario | Distribution of training animals across generations | Number of generations in training | Number of animals in training | Prediction accuracy (±SD) |
| --- | --- | --- | --- | --- |
| 1 | G9=250 | 1 | 250 | 0.37±0.0085 |
| 2 | G9=G8=250 | 2 | 500 | 0.52±0.0046 |
| 3 | G9=G8=G7=250 | 3 | 750 | 0.53±0.0063 |
| 4 | G9=125 | 1 | 125 | 0.24±0.083 |
| 5 | G9=G8=125 | 2 | 250 | 0.38±0.073 |
| 6 | G9=G8=G7=125 | 3 | 375 | 0.40±0.068 |
| 7 | G9=G8=G7=G6=125 | 4 | 500 | 0.47±0.055 |
| 8 | G9=G8=G7=G6=G5=125 | 5 | 625 | 0.46±0.052 |
| 9 | G9=G8=G7=G6=G5=G4=125 | 6 | 750 | 0.50±0.051 |

1. lEW

| Scenario | Distribution of training animals across generations | Number of generations in training | Number of animals in training | Prediction accuracy (±SD) |
| --- | --- | --- | --- | --- |
| 1 | G9=250 | 1 | 250 | 0.37±0.037 |
| 2 | G9=G8=250 | 2 | 500 | 0.52±0.031 |
| 3 | G9=G8=G7=250 | 3 | 750 | 0.58±0.031 |
| 4 | G9=125 | 1 | 125 | 0.24±0.087 |
| 5 | G9=G8=125 | 2 | 250 | 0.34±0.075 |
| 6 | G9=G8=G7=125 | 3 | 375 | 0.46±0.089 |
| 7 | G9=G8=G7=G6=125 | 4 | 500 | 0.50±0.065 |
| 8 | G9=G8=G7=G6=G5=125 | 5 | 625 | 0.52±0.061 |
| 9 | G9=G8=G7=G6=G5=G4=125 | 6 | 750 | 0.54±0.042 |

1. lBW

| Scenario | Distribution of training animals across generations | Number of generations in training | Number of animals in training | Prediction accuracy (±SD) |
| --- | --- | --- | --- | --- |
| 1 | G9=250 | 1 | 250 | 0.69±0.010 |
| 2 | G9=G8=250 | 2 | 500 | 0.72±0.011 |
| 3 | G9=G8=G7=250 | 3 | 750 | 0.73±0.018 |
| 4 | G9=125 | 1 | 125 | 0.47±0.077 |
| 5 | G9=G8=125 | 2 | 250 | 0.63±0.090 |
| 6 | G9=G8=G7=125 | 3 | 375 | 0.67±0.022 |
| 7 | G9=G8=G7=G6=125 | 4 | 500 | 0.65±0.016 |
| 8 | G9=G8=G7=G6=G5=125 | 5 | 625 | 0.68±0.031 |
| 9 | G9=G8=G7=G6=G5=G4=125 | 6 | 750 | 0.69±0.024 |

1. lAH

| Scenario | Distribution of training animals across generations | Number of generations in training | Number of animals in training | Prediction accuracy (±SD) |
| --- | --- | --- | --- | --- |
| 1 | G9=250 | 1 | 250 | 0.26±0.034 |
| 2 | G9=G8=250 | 2 | 500 | 0.29±0.022 |
| 3 | G9=G8=G7=250 | 3 | 750 | 0.34±0.026 |
| 4 | G9=125 | 1 | 125 | 0.20±0.074 |
| 5 | G9=G8=125 | 2 | 250 | 0.25±0.11 |
| 6 | G9=G8=G7=125 | 3 | 375 | 0.25±0.11 |
| 7 | G9=G8=G7=G6=125 | 4 | 500 | 0.25±0.11 |
| 8 | G9=G8=G7=G6=G5=125 | 5 | 625 | 0.25±0.099 |
| 9 | G9=G8=G7=G6=G5=G4=125 | 6 | 750 | 0.26±0.086 |

1. lYW

| Scenario | Distribution of training animals across generations | Number of generations in training | Number of animals in training | Prediction accuracy (±SD) |
| --- | --- | --- | --- | --- |
| 1 | G9=250 | 1 | 250 | 0.24±0.0097 |
| 2 | G9=G8=250 | 2 | 500 | 0.20±0.016 |
| 3 | G9=G8=G7=250 | 3 | 750 | 0.22±0.016 |
| 4 | G9=125 | 1 | 125 | 0.24±0.054 |
| 5 | G9=G8=125 | 2 | 250 | 0.24±0.060 |
| 6 | G9=G8=G7=125 | 3 | 375 | 0.23±0.11 |
| 7 | G9=G8=G7=G6=125 | 4 | 500 | 0.22±0.10 |
| 8 | G9=G8=G7=G6=G5=125 | 5 | 625 | 0.23±0.092 |
| 9 | G9=G8=G7=G6=G5=G4=125 | 6 | 750 | 0.24±0.084 |

1. lPD

| Scenario | Distribution of training animals across generations | Number of of generations in training | Number of animals in training | Prediction accuracy (±SD) |
| --- | --- | --- | --- | --- |
| 1 | G9=250 | 1 | 250 | 0.34±0.0076 |
| 2 | G9=G8=250 | 2 | 500 | 0.35±0.012 |
| 3 | G9=G8=G7=250 | 3 | 750 | 0.38±0.0042 |
| 4 | G9=125 | 1 | 125 | 0.19±0.11 |
| 5 | G9=G8=125 | 2 | 250 | 0.21±0.12 |
| 6 | G9=G8=G7=125 | 3 | 375 | 0.26±0.083 |
| 7 | G9=G8=G7=G6=125 | 4 | 500 | 0.26±0.083 |
| 8 | G9=G8=G7=G6=G5=125 | 5 | 625 | 0.28±0.070 |
| 9 | G9=G8=G7=G6=G5=G4=125 | 6 | 750 | 0.27±0.087 |

(15) lPS

| Scenario | Distribution of training animals across generations | Number of generations in training | Number of animals in training | Prediction accuracy (±SD) |
| --- | --- | --- | --- | --- |
| 1 | G9=250 | 1 | 250 | 0.10±0.047 |
| 2 | G9=G8=250 | 2 | 500 | 0.20±0.047 |
| 3 | G9=G8=G7=250 | 3 | 750 | 0.21±0.041 |
| 4 | G9=125 | 1 | 125 | 0.060±0.063 |
| 5 | G9=G8=125 | 2 | 250 | 0.061±0.12 |
| 6 | G9=G8=G7=125 | 3 | 375 | 0.10±0.093 |
| 7 | G9=G8=G7=G6=125 | 4 | 500 | 0.12±0.083 |
| 8 | G9=G8=G7=G6=G5=125 | 5 | 625 | 0.11±0.066 |
| 9 | G9=G8=G7=G6=G5=G4=125 | 6 | 750 | 0.10±0.065 |
