# Supplementary figures and images for: Effects of number of training generations on genomic prediction for various traits in a layer chicken population
Source: Genet Sel Evol. 2016 Mar 19;48:22. doi: 10.1186/s12711-016-0198-9 (PMC4799631; doi:10.1186/s12711-016-0198-9)

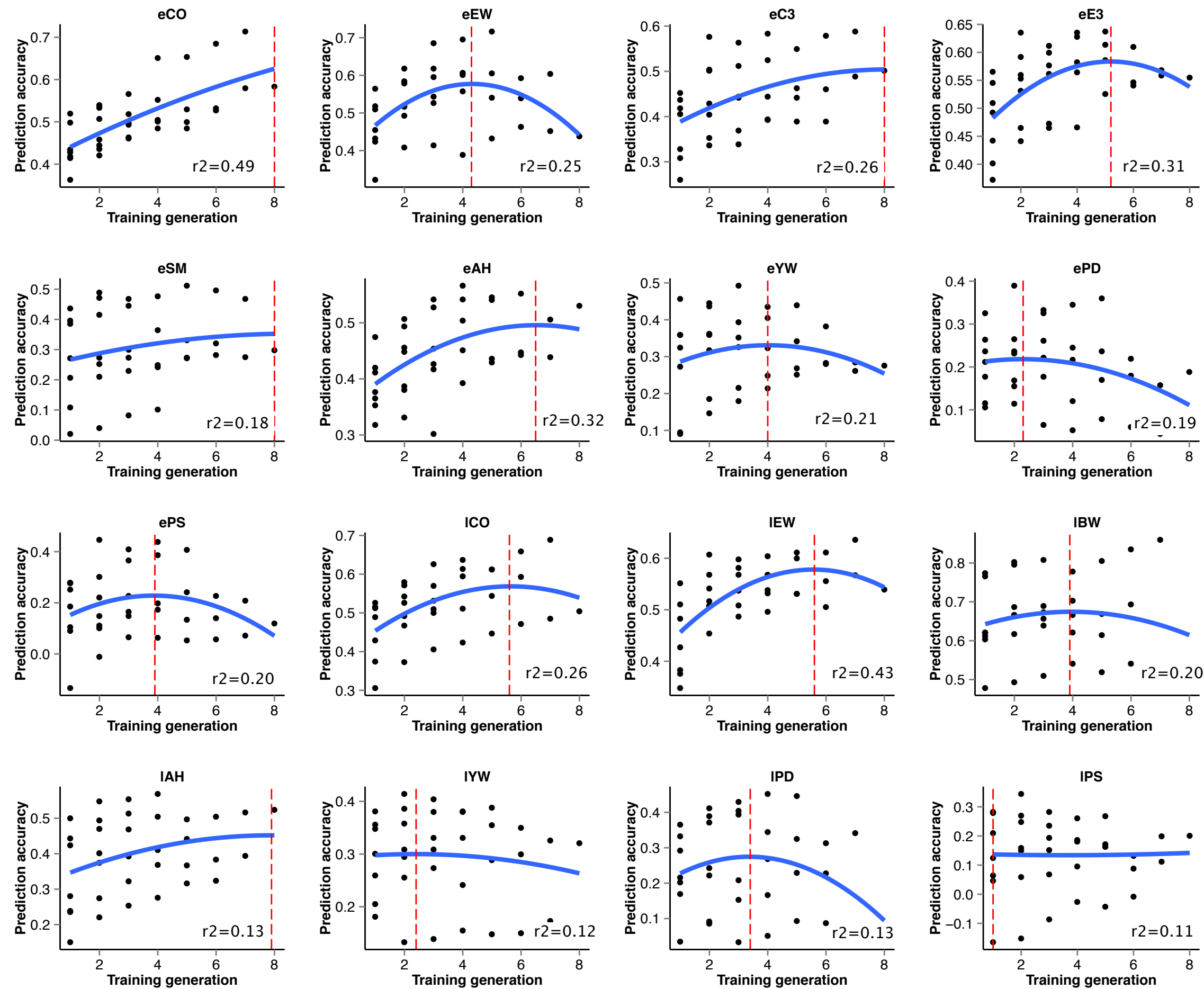

Supplement: Supplementary file 2 — 10.1186/s12711-016-0198-9 Scatter plot of accuracies of genomic predictions across different validation sets over training generations for each trait. The blue line is the regression of the accuracy on the number of training generations. The red line indicates the optimal number of training generations. The R-squared of regression line is presented as r2. [file 12711_2016_198_MOESM2_ESM.pdf]
